# Supplementary material for: Integrating deep learning CT-scan model, biological and clinical variables to predict severity of COVID-19 patients
Source: Nat Commun. 2021 Jan 27;12:634. doi: 10.1038/s41467-020-20657-4 (PMC7840774; doi:10.1038/s41467-020-20657-4)
Supplement: Supplementary file 1 — Supplementary Information [file 41467_2020_20657_MOESM1_ESM.pdf]

Supplementary material of “Integrating deep learning CT-scan model, biological and clinical variables to predict severity of COVID-19 patients”

Supplementary Figures

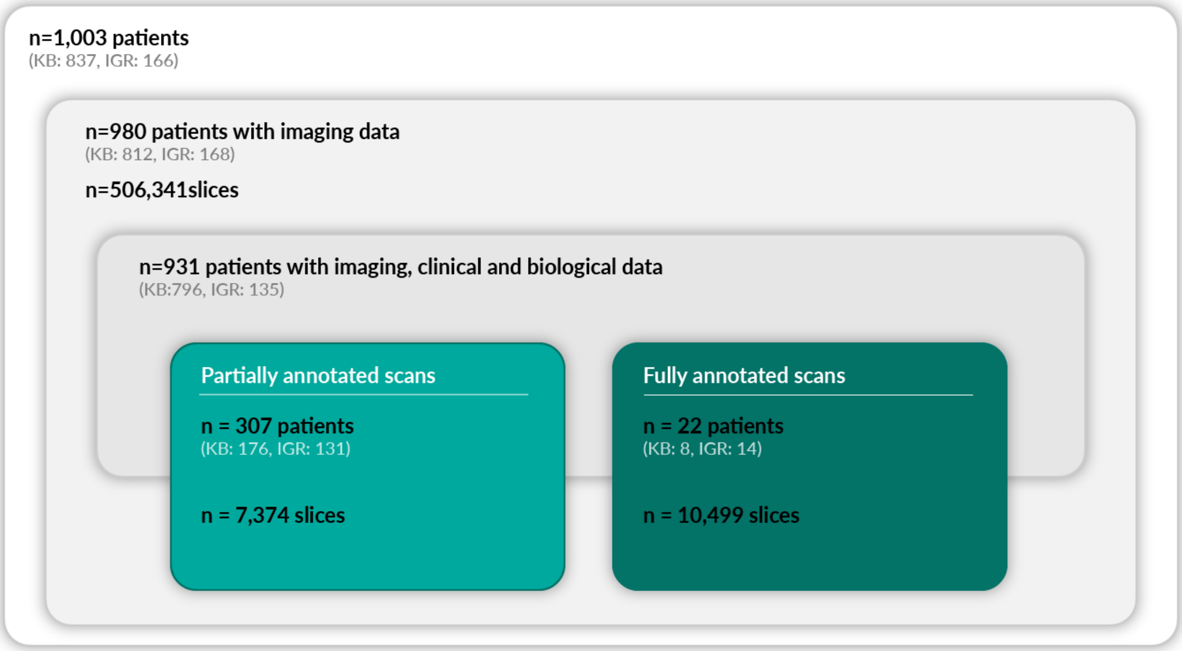

Supplementary Fig. 1: Description of the retrospective cohort. We provide information on the collected data per hospital for all patients.

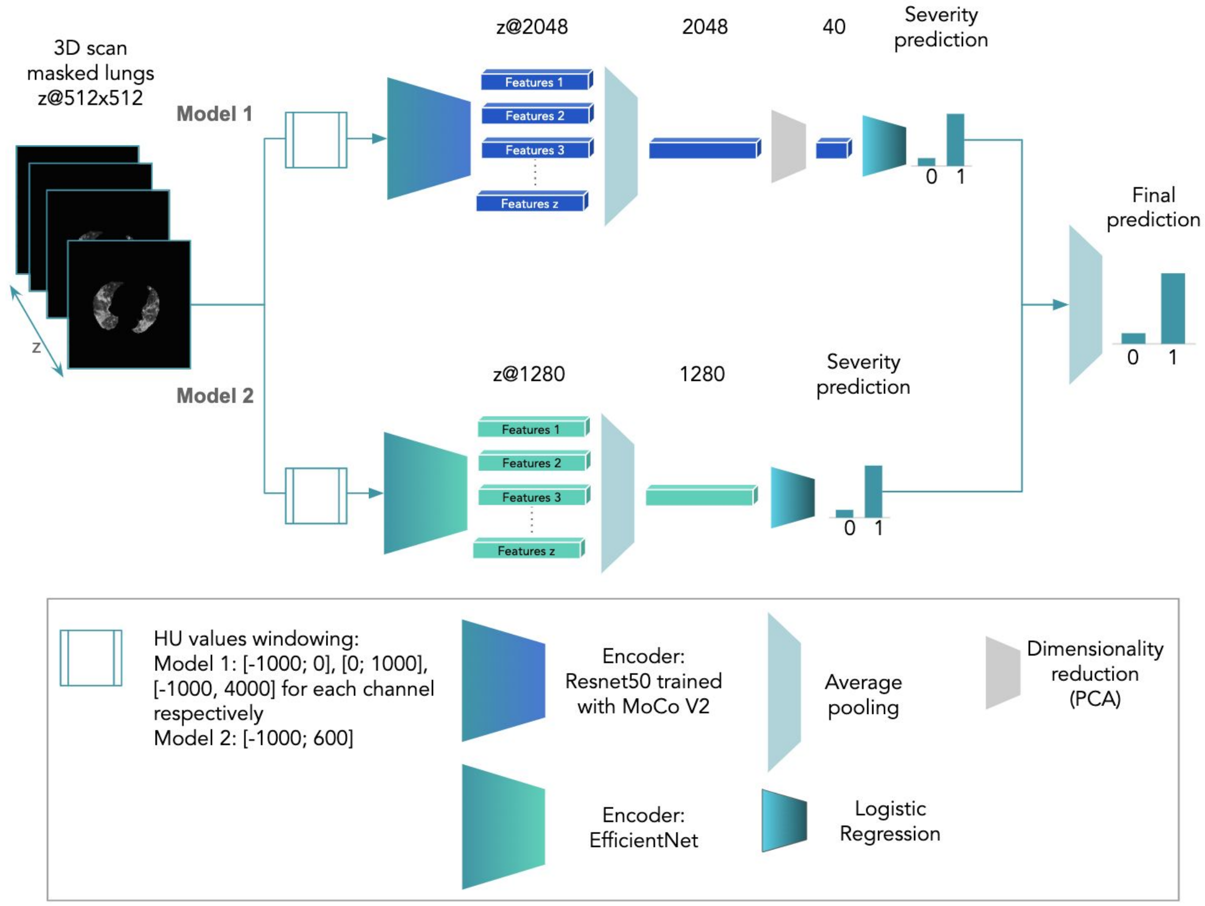

Supplementary Fig. 2: Neural network to predict severity from 3D chest CT scans. The final prediction of the network is one of the 6 variables of the *AI-severity* score. Two different pipelines were used: one using Resnet50 (trained with MocoV2 on 1 million public CT scan slices) as encoder (model 1) and one using EfficientNet B0 as encoder (model 2).

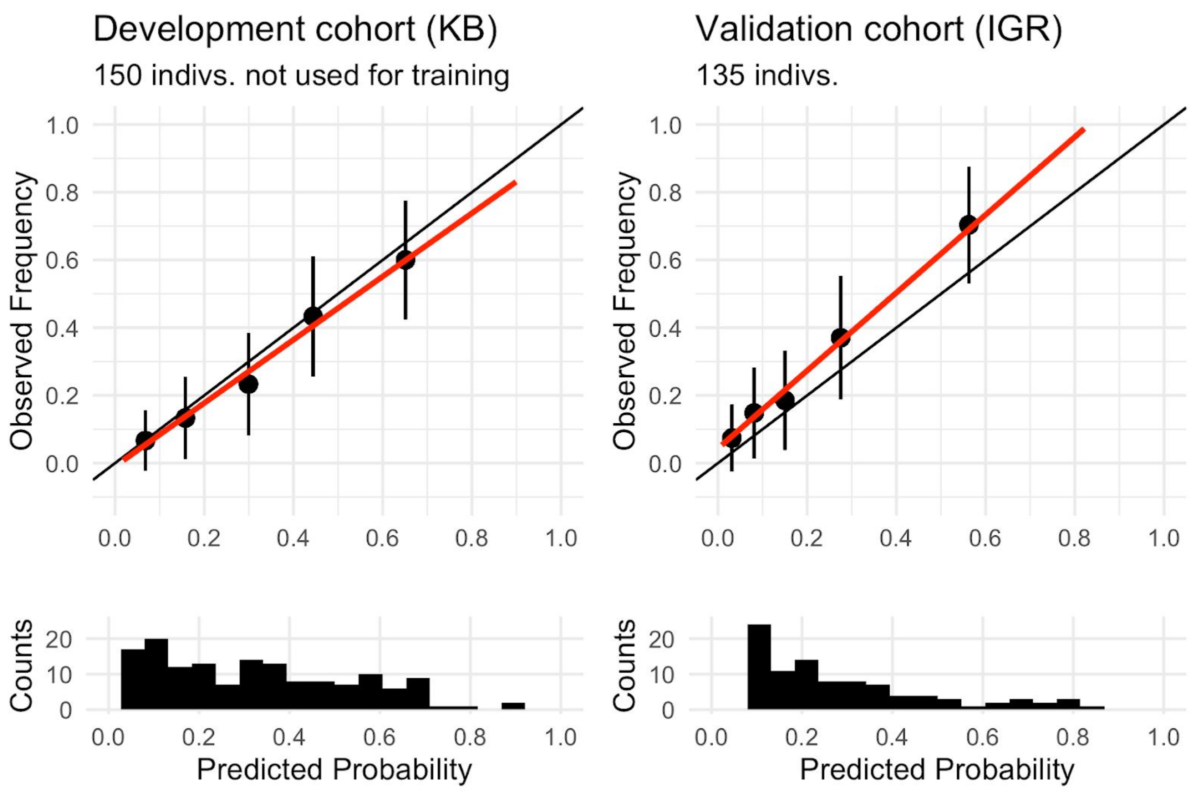

Supplementary Fig. 3: Calibration plot. We considered five bins with the same number of patients to compare predicted probabilities provided by *AI-severity* and observed frequencies. We used the quantiles of the predicted probabilities as provided by *AI-severity* to construct bins. 95% confidence intervals were obtained using non-parametric bootstrap. Lines in red were obtained using linear regression by regressing the true outcome with the predicted probability as a predictor variable. The histogram displays the distribution of *AI-severity* for the hospitalized patients. The code to generate the figure was excerpted from the webpage https://darrendahly.github.io/post/homr/.

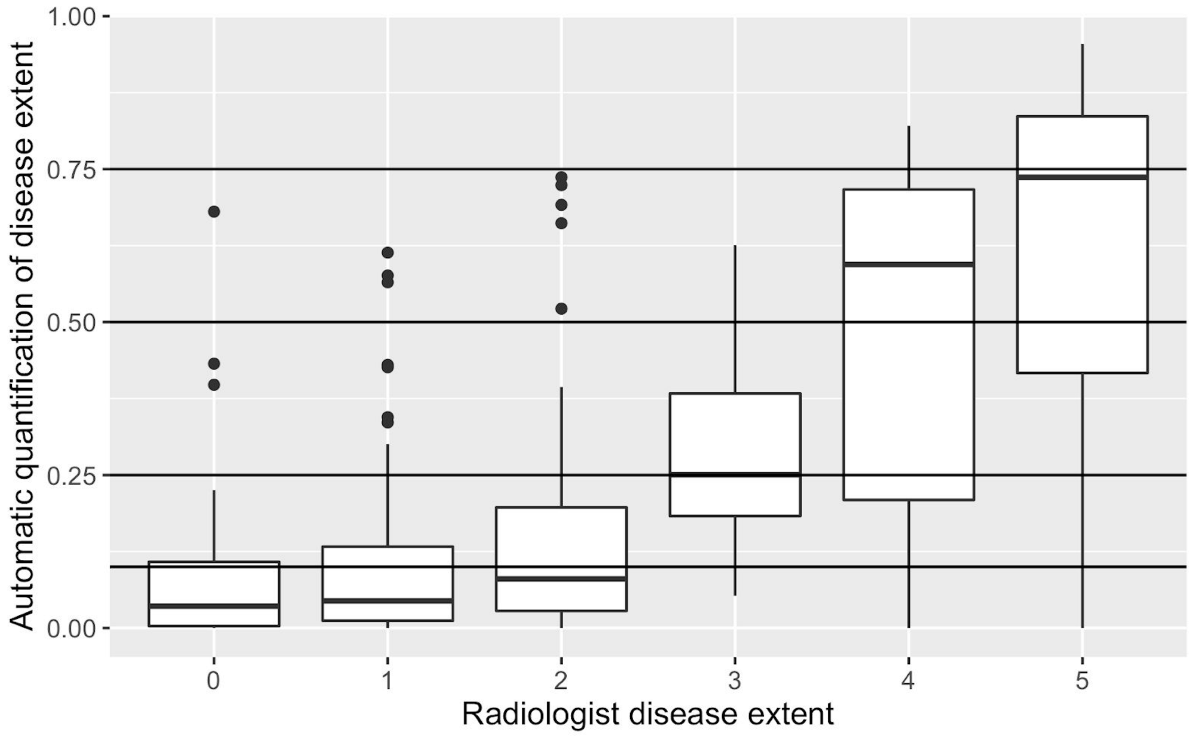

Supplementary Fig. 4: Boxplot to compare automatic quantification of disease extent using a neural network segmentation model and disease extent as quantified by a radiologist. The coding of disease extent in the radiologist report is as follows: 0 (0% of lesions), 1 (<10% of lesions), 2 (between 10 and 25% of lesions), 3 (between 25 and 50% of lesions), 4 (between 50 and 75% of lesions), 5 (more than 75% of lesions). The lower and upper hinges correspond to the first and third quartiles. The upper whisker extends from the hinge to the largest value no further than 1.5 * IQR from the hinge (where IQR is the inter-quartile range). The lower whisker extends from the hinge to the smallest value at most 1.5 * IQR of the hinge. Data beyond the end of the whiskers are called "outlying" points and are plotted individually. The sample sizes for each boxplot are as follows: n=22 (disease extent=0), n=54 (disease extent=1), n=38 (disease extent=2), n=11 (disease extent=3), n=15 (disease extent=4), n=13 (disease extent=5).

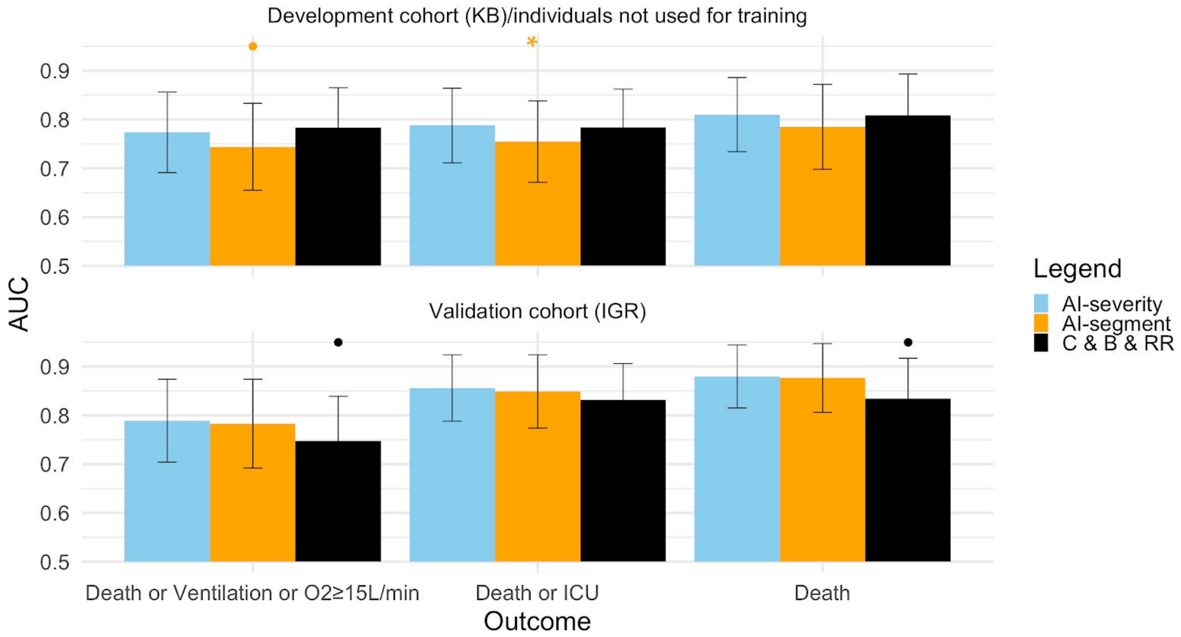

Supplementary Fig. 5: AUC values when comparing *AI-severity* to *AI-segment* and to the model including Clinical, Biological variables and disease extent extracted from a Radiologic Report *(C & B & RR)*. All three models were trained using the severity outcome defined as an oxygen flow rate of 15 L/min or higher, the need for mechanical ventilation, or death. When evaluating the three models on the alternative outcomes, models were not trained again. AUC results are reported on the leftover KB patients from the development cohort (150 patients) and the external validation set from IGR (135 patients). Error bars represent the 95% confidence intervals. Stars indicate the order of magnitude of p-values for the DeLong one-sided test in which we test if $AUC_{AI\text{-}severity} > AUC_{other\ score}$, *• 0.05<p≤0.10, * 0.01<p≤0.O5, ** 0.001<p≤0.01, *** p≤0.001.*

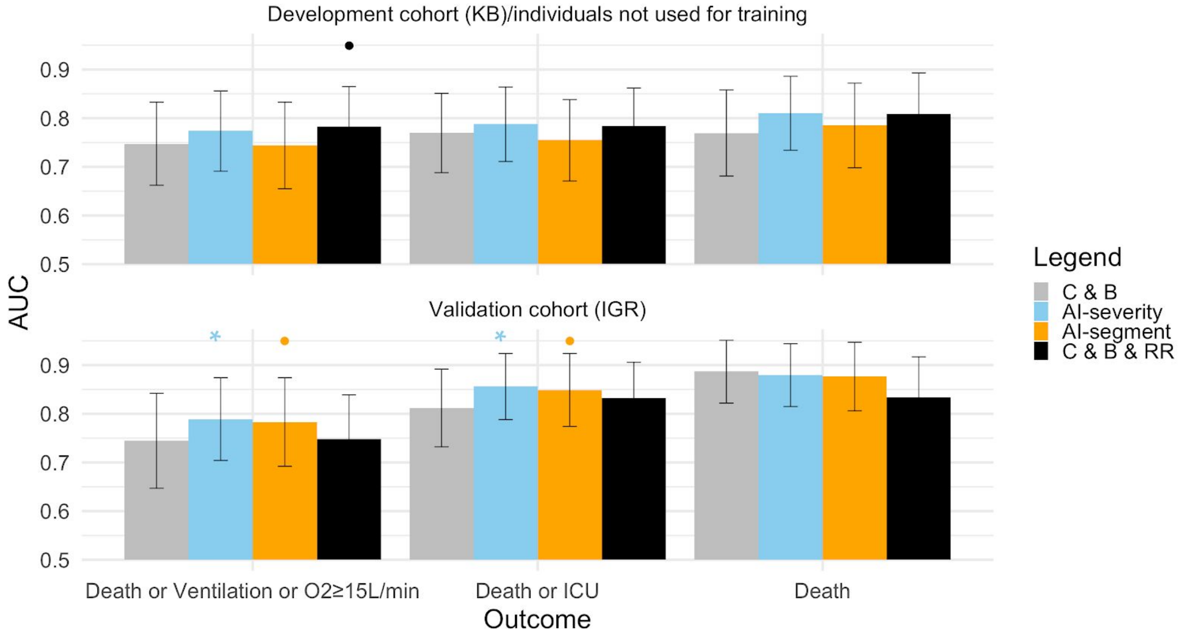

Supplementary Fig. 6: AUC values when comparing the model that includes Clinical, and Biological variables (*C & B*) to the three models that additionally include CT-scan information (*AI-severity*, *C & B & RR*, *AI-segment*). All four models were trained using the severity outcome defined as an oxygen flow rate of 15 L/min or higher, the need for mechanical ventilation, or death. When evaluating the three models on the alternative outcomes, models were not trained again. AUC results are reported on the leftover KB patients from the development cohort (150 patients) and the external validation set from IGR (135 patients). Error bars represent the 95% confidence intervals. Stars indicate the order of magnitude of p-values for the DeLong one-sided test in which we test $AUC_{C\ \&\ B} < AUC_{other\ score}$, • *0.05<p≤0.10, * 0.01<p≤0.05, ** 0.001<p≤0.01, *** p≤0.001.*

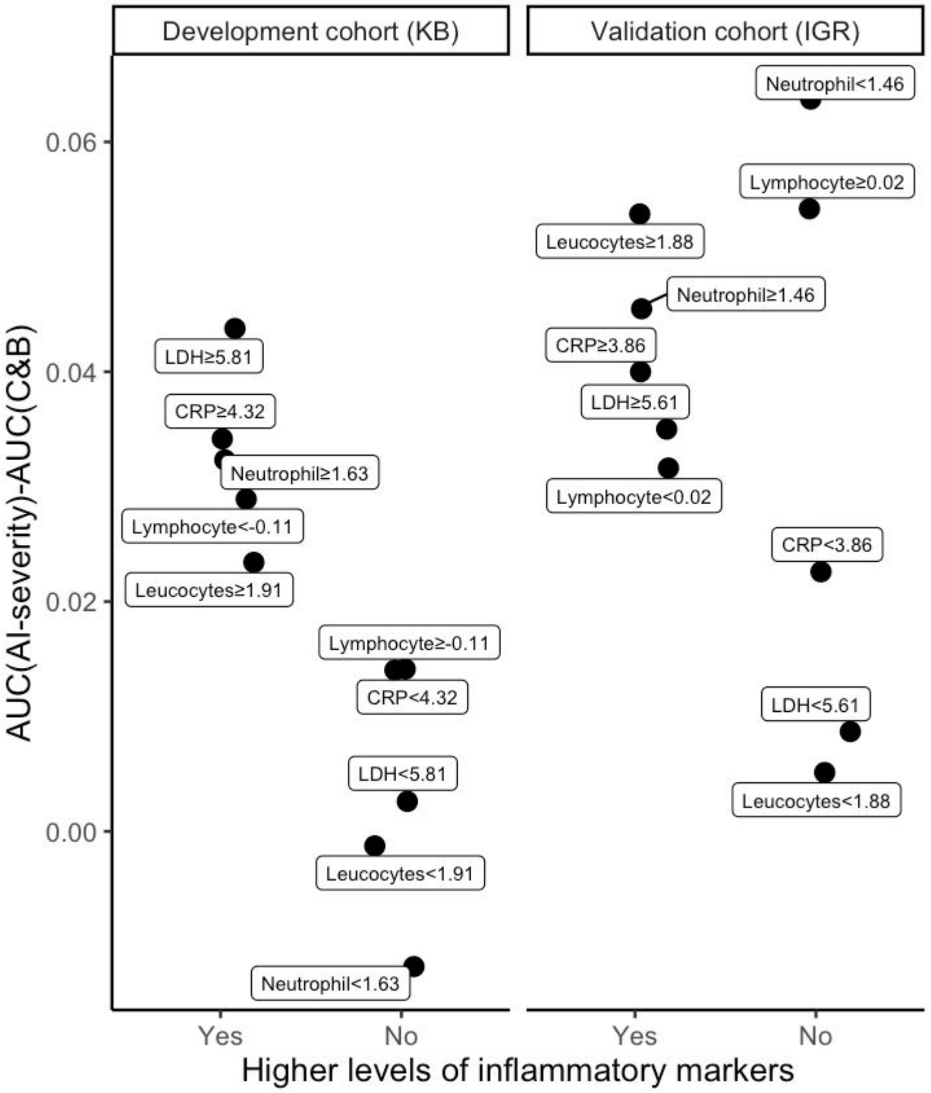

Supplementary Fig. 7: Point estimates of differences of AUC when comparing *AI-severity*, which includes CT-scan information in addition to clinical and biological variables, to *C & B*, which includes clinical and biological variables only. For each variable, we consider two subgroups whether or not the patient value is above or below the median value. CRP, LDH, Neutrophil and Leukocytes are variables for which higher values indicate higher inflammation whereas higher inflammatory response corresponds to lower values of lymphocyte counts. For the development cohort (KB), there are 150 leftover individuals that were not used during training and for the validation cohort (IGR), there are 135 individuals.

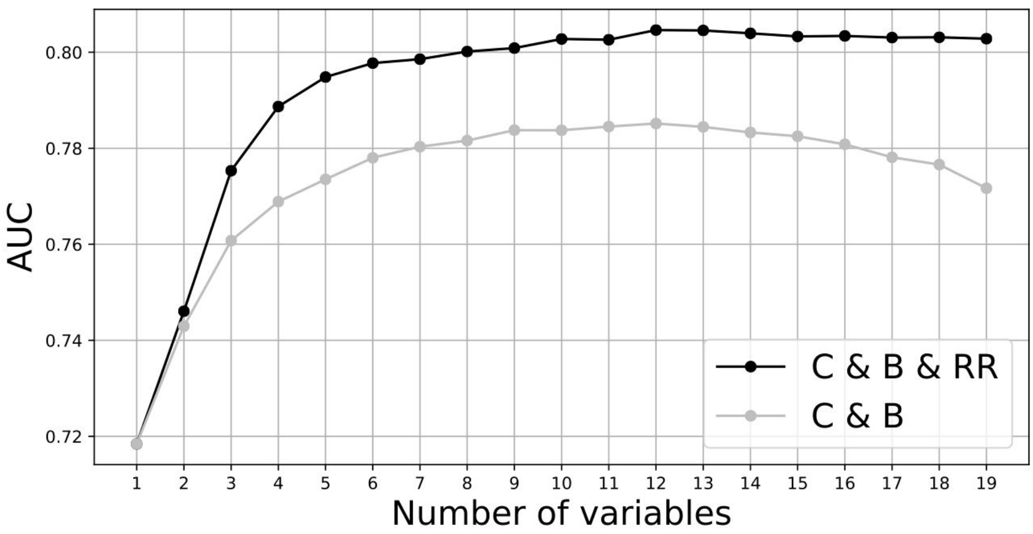

Supplementary Fig. 8: AUC curve as a function of the number of clinical and biological information added to the multimodal model. Variables included in the models consist of CT scan variables only and then a greedy algorithm adds clinical or biological variables iteratively. At each step of the algorithm, the variable that results in the largest increase of AUC score is added.

Supplementary Tables

Variable	AUC for the 150 leftover patients of the KB development cohort	AUC for the 135 IGR patients of the validation cohort
Age > 60	0.884 (0.828 - 0.940)	0.786 (0.710 - 0.862)
Sex	0.933 (0.892 - 0.975)	0.893 (0.838 - 0.947)
Oxygen saturation > 90	0.761 (0.681 - 0.840)	0.782 (0.676 - 0.888)
Disease extent > 2	0.926 (0.887 - 0.965)	0.881 (0.819 -0.943)
Crazy paving	0.775 (0.700 - 0.851)	0.725 (0.637 - 0.812)
Condensation	0.6365 (0.534 - 0.737)	0.675 (0.583 -0.767)
GGO	0.800 (0.655 - 0.944)	0.583 (0.475 - 0.690)

Supplementary Table 1: *AI-severity* model performances on other classification tasks than severity prediction. AUC scores are reported on both KB and IGR validation sets when re-training the *AI-severity* model to predict a few clinical and radiological variables we have selected. To retrain the model, we considered the last hidden layer of *AI-severity* as a feature vector and used logistic regression to predict clinical/radiological outcome.

Variable	Coding/unit	Transformation	Coefficient
Oxygen saturation	%	-log(1 + 100 - X)	-0.569
Neural network variable		None	0.769
Age	year	None	0.0121
Sex	1 for male<br>0 for female	None	0.412
Platelet	G/L	log(0.001 + X)	-0.567
Urea	mmol/L	log(0.001 + X)	0.393

Supplementary Table 2: Coefficients, transformation, and units to compute the *AI-severity* score.

Model description	KB	IGR	KB CV
O2 ≥15L/min or Ventilation or Death			
AI-severity	0.774 (0.691-0.856)	**0.789 (0.704-0.874)**	**0.793 (0.689-0.881)**
Neural network analysis	0.763 (0.674-0.851)	0.748 (0.657-0.839)	0.748 (0.627-0.839)
C & B	0.747 (0.662-0.833)	0.745 (0.647-0.842)	0.776 (0.686-0.871)
C & B & RR	**0.783 (0.702-0.865)**	0.748 (0.658-0.839)	**0.793 (0.713-0.899)**
AI-segment	0.744 (0.655-0.833)	0.783 (0.692-0.874)	0.787 (0.685-0.881)
MIT analytics	0.703 (0.611-0.796)	0.615 (0.508-0.722)	
CALL	0.642 (0.549-0.735)	0.582 (0.482-0.681)	
Colombi et al.	0.695 (0.603-0.787)	0.554 (0.452-0.656)	
Colombi et al. (with CT scan)	0.702 (0.613-0.792)	0.561 (0.456-0.666)	
COVID_GRAM	0.716 (0.631-0.802)	0.713 (0.610-0.817)	
CURB65	0.701 (0.610-0.792)	0.674 (0.579-0.770)	
Yan et al.	0.694 (0.606-0.782)	0.604 (0.508-0.700)	
NEWS2_carr	0.624 (0.526-0.722)	0.716 (0.619-0.813)	
NEWS2 for COVID-19	0.698 (0.606-0.790)	0.760 (0.672-0.848)	
4C mortality	0.702 (0.611-0.792)	0.661 (0.559-0.763)	
Liang et al.	0.676 (0.575-0.777)	0.652 (0.549-0.756)	
Death or ICU			
AI-severity	**0.788 (0.711-0.864)**	**0.856 (0.788-0.924)**	0.793 (0.708-0.890)
Neural network analysis	0.767 (0.685-0.849)	0.825 (0.748-0.902)	0.749 (0.632-0.857)
C & B	0.770 (0.688-0.851)	0.812 (0.732-0.892)	0.782 (0.679-0.878)
C & B & RR	0.784 (0.706-0.862)	0.832 (0.757-0.906)	**0.794 (0.714-0.892)**
AI-segment	0.755 (0.671-0.838)	0.849 (0.774-0.924)	0.792 (0.715-0.900)
MIT analytics	0.703 (0.614-0.791)	0.660 (0.564-0.756)	
CALL	0.598 (0.503-0.693)	0.604 (0.504-0.704)	
Colombi et al.	0.670 (0.579-0.761)	0.587 (0.486-0.687)	
Colombi et al. (with CT scan)	0.651 (0.558-0.745)	0.614 (0.510-0,718)	

COVID_GRAM	0.708 (0.621-0.795)	0.713 (0.611-0.814)	
CURB65	0.688 (0.598-0.778)	0.709 (0.618-0.800)	
Yan et al.	0.717 (0.634-0.800)	0.675 (0.582-0.767)	
NEWS2_carr	0.626 (0.531-0.720)	0.739 (0.647-0.830)	
NEWS2 for COVID-19	0.716 (0.630-0.802)	0.790 (0.713-0.868)	
4C mortality	0.704 (0.615-0.792)	0.684 (0.588-0.780)	
Liang et al.	0.709 (0.613-0.804)	0.744 (0.653-0.835)	
Death			
AI-severity	0.810 (0.734-0.886)	**0.880 (0.815-0.944)**	0.787 (0.718-0.902)
Neural network analysis	0.752 (0.660-0.845)	0.753 (0.644-0.862)	0.710 (0.630-0.835)
C & B	0.769 (0.681-0.858)	0.887 (0.822-0.951)	**0.794 (0.683-0.914)**
C & B & RR	0.809 (0.726-0.893)	0.834 (0.752-0.917)	0.786 (0.685-0.924)
AI-segment	0.785 (0.698-0.872)	0.877 (0.806-0.947)	0,782 (0.711-0.916)
MIT analytics	**0.818 (0.729-0.907)**	0.660 (0.546-0.773)	
CALL	0.707 (0.604-0.810)	0.596 (0.484-0.709)	
Colombi et al.	0.779 (0.689-0.869)	0.579 (0.462-0.697)	
Colombi et al. (with CT scan)	0.772 (0.688-0.856)	0.498 (0.372-0.625)	
COVID_GRAM	0.791 (0.700-0.882)	0.789 (0.690-0.888)	
CURB65	0.814 (0.736-0.888)	0.700 (0.592-0.808)	
Yan et al.	0.637 (0.532-0.743)	0.707 (0.600-0.814)	
NEWS2_carr	0.641 (0.536-0.746)	0.641 (0.516-0.767)	
NEWS2 for COVID-19	0.746 (0.656-0.836)	0.762 (0.670-0.854)	
4C mortality	0.807 (0.729-0.886)	0.739 (0.640-0.838)	
Liang et al.	0.605 (0.479-0.730)	0.757 (0.629-0.885)	

Supplementary Table 3: AUC values for the different models on the different sets. Each model (Neural network analysis, *AI-severity*, *C & B*, *C & B & RR*, *AI-segment*) was trained on 646 patients from KB. Results are reported on the leftover 150 patients from the development KB cohort and for the 135 patients from the IGR validation set. For the models we trained, we also report performance obtained using 5 fold cross validation stratified by outcome and age (CV KB). For each outcome, the best models are highlighted in boldface. C & B: Clinical and Biological variables, C & B & RR: Clinical and Biological variables and the ones of the Radiological Report.

	Correlation	Lower 95% C.I.	Upper 95% C.I.
Disease extent	0.62	0.58	0.67
Oxygen saturation	-0.53	-0.58	-0.48
LDH	0.46	0.39	0.52
CRP	0.43	0.37	0.49
Age	0.3	0.24	0.36
Respiratory rate	0.25	0.17	0.32
Neutrophil	0.25	0.18	0.31
Leucocytes	0.22	0.15	0.29
Urea	0.18	0.11	0.25
Ferritin	0.15	0.03	0.26
Diastolic pressure	-0.15	-0.22	-0.07
BMI	0.14	0.04	0.23
Total bilirubin	0.11	0.03	0.18
Platelet	0.09	0.02	0.16
Weight	0.08	-0.01	0.16
Creatine kinase	0.07	-0.01	0.15
Haemoglobin	-0.06	-0.14	0.01
Body temperature	0.06	-0.01	0.13
Cardiac frequency	-0.06	-0.13	0.02
Conjugated bilirubin	-0.05	-0.32	0.23
Lymphocyte	-0.04	-0.11	0.03
Systolic pressure	-0.04	-0.11	0.03
Symptoms duration before examination	-0.04	-0.11	0.03
Monocyte	-0.02	-0.09	0.05
Height	-0.01	-0.11	0.09

Supplementary Table 4: Correlation of clinical and biological variables with the prognosis obtained with a weakly-supervised neural network. Correlation was computed using 817 patients from the KB hospital. Variables are sorted in decreasing order when considering the squared correlation value for ranking.

Models	Variables included									
1. AI-severity<br>*2. AI-segment*<br>3. Clinical & bio & Radiological Report (*C & B & RR*)	Oxygen saturation	Disease extent	Age	Sex	Platelet	Urea				
Clinical and bio (C & B)	Oxygen saturation	Age	Sex	LDH	Platelet	Chronic kidney disease	Dyspnea	Hypertension	Neutrophil	Urea

Supplementary Table 5: Names of the variables included in the different models.

Score	Variable	Value used
CALL score	Comorbidity	1 if any of cardiac disease, asthma, emphysema, diabetes, or hypertension else 0
Colombi et al.	Cardiovascular disease	1 if any of cardiac disease or hypertension else 0
COVID-Gram and Liang et al.	XRay abnormality	1 if any lesion is observed on the CT scan, else 0
COVID-gram	Hemoptysis	0
COVID-gram, NEWS2	Unconsciousness	0
COVID-gram	Number of comorbidities	Count of cardiac disease, asthma, diabetes, emphysema, chronic kidney disease, hypertension
Liang et al.	Number of comorbidities	Count of cardiac disease, diabetes, emphysema, chronic kidney disease, cancer, hypertension
Liang et al.	Chronic obstructive pulmonary disease	Same value as emphysema
4C	Number of comorbidities	Count of cardiac disease, diabetes, emphysema, chronic kidney disease, cancer
4C	Glasgow coma score	0
NEWS2	Air or oxygen	0
NEWS2	Oxygen liters	0
NEWS2 for COVID	Estimated Glomerular Filtration Rate	0
CURB-65	Confusion	0

Supplementary Table 6: imputed values for the missing or partially-missing variables of the severity/mortality scores for COVID-19 patients. We used 0 for imputation as the missing variables are included in linear models only and the constant value does not impact AUC.

Supplementary Notes

Annotation scenario of CT scans by radiologists

Two radiologists (4 and 9 years of experience) examined and annotated 307 anonymized chest scans independently and without access to the patient's clinic or COVID-19 PCR results. All CT images were viewed with lung window parameters (width, 1500 HU; level, -550 HU) using the SPYD software developed by Owkin. Regions of interest were annotated by the radiologists in four distinct classes: healthy pulmonary parenchyma, ground glass opacity, consolidation, crazy-paving. The presence of organomegaly was also notified when present, as a binary class. When multiple CT images were available for a single patient, the image to analyze was selected using the SPYD software. One AI and imaging PhD student also provided full 3D annotation of the four classes on 22 anonymized chest scans using the 3D Slicer software.

Method to segment CT-scans

The model used to perform segmentation and compute the AI-*segment* score was based on 3 segmentation networks: 3D Resnet50 (Hara, Kataoka, and Satoh 2017) , 2.5D U-Net, and 2D U-Net (Ronneberger, Fischer, and Brox 2015). U-Net consists of convolution, max pooling, ReLU activations, concatenation and up-sampling layers with sections: contraction, bottleneck, and expansion (Supplementary Fig. 9). ResNet contains convolutions, max pooling, batch normalization, and ReLU layers that are grouped in multiple bottleneck blocks. All models were trained on CT scans provided by Kremlin-Bicêtre (KB) and evaluated on annotated CT scans from Institut Gustave Roussy (IGR). The dataset was divided into two categories: Fully Annotated Scans (FAS) composed of 22 scans (8 from KB and 14 from IGR) and Partially Annotated Scans (PAS) composed of 307 scans (176 from KB and 131 from IGR). PAS contains a total of 7,374 annotated slices and 24,476,521 annotated pixels, i.e. 24 slices per PAS and 3,319 pixels annotated per slice on average.

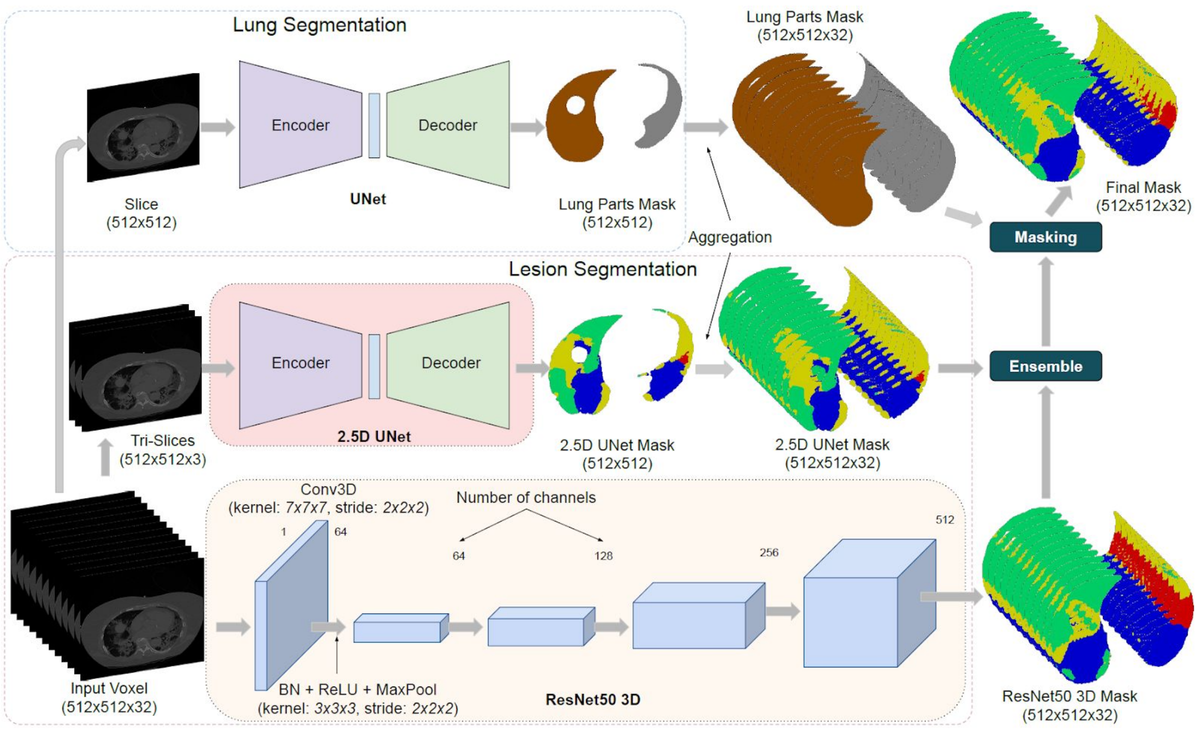

Supplementary Fig. 9: architecture of the segmentation model. Proposed pipeline to generate lesion volumetry estimates from patient CT scans employing ensemble of segmentation networks. Normalized patient scans are provided to our trained 2.5D U-Net and 3D ResNet50. The masks predicted from both models are then merged by arithmetic mean. In parallel, we segment left-right lungs from the patient scans using a dedicated U-Net. Finally, the left-right lung mask is used to mask-out lesions in left and right lungs from the ensemble output. This pipeline utilizes the complementary features learned by a weak model (2.5D U-Net) and a strong one (3D ResNet50).

2D U-Net was trained for left/right lung segmentation and 3D ResNet and 2.5D U-Net were used for lesion segmentation. 3D ResNet50 was trained on 8 KB FAS (i.e. 3,704 slices). Inputs for the 3D ResNet consist of a height and a width of 128, and a depth of 32. We initialized the 3D ResNet with pretrained weights (Chen, Ma, and Zheng 2019). We then trained the network with Stochastic Gradient Descent for parameter optimization and an initial learning rate of 0.1 with a decay factor of 0.1 every 20 epochs. The network was trained for a total of 100 epochs. For the 2.5D U-Net, we first pretrained the network on a left-right lung segmentation task using the LCTCS dataset (Yang et al. 2018). The network was then trained on the KB dataset using Adam optimization algorithm with a learning rate, weight decay, gradient clipping and learning rate decay parameters of 1e-3, 1e-8, 1e-1, and 0.1 (applied at epochs 90 and 150) for 300 epochs. While the validation set remains the same as when evaluating the 3D resnet50 model, 176 KB PAS scans were added to the 8 KB FAS, in the training set. PAS were only added to the 2.5D U-Net training set due to the incompleteness of the annotated volume in the scans which would not satisfy the volumetric

requirements of the 3D ResNet50 input. Finally, for the left/right lung segmentation, the 2D U-Net was trained on the 8 KB FAS. Similarly to 2.5D U-Net, Adam optimization algorithm was used with a learning rate, weight decay, gradient clipping, learning rate decay, and number of epochs of 1e-3, 1e-8, 1e-1, 0.1 (applied at epoch 70), and 104. Both 2.5D U-Net and 2D U-Net used affine transformation and contrast change for data augmentation while 3D ResNet50 used affine transformation, contrast change, thin plate splines, and flipping. 3D ResNet and 2.5D U-Net are trained through the minimization of a cross entropy loss and 2D U-Net minimized a binary cross entropy loss. All training was performed on NVIDIA Tesla V100 GPUs using Pytorch as a coding framework. During the validation phase, ensemble inference (Baldeon Calisto and Lai-Yuen 2020) was performed on all available scans by averaging lesion masks, which were predicted from the 3D ResNet and 2.5D U-Net models, using arithmetic mean.

We evaluated the segmentation model on three distinct aspects. First, we evaluated its ability to perform accurate segmentation. To this aim, we computed F1 scores for the PAS (partially annotated scans) and FAS (fully annotated scans), of the IGR test set, when discriminating lesions versus sane areas inside the lung. Micro-averaging was used to limit the effect of class imbalance for the three different lesion types. We also reported the accuracy to discriminate background versus lung regions using FAS where background regions outside of the lung were annotated. Second, we evaluated its ability to estimate the proportion of each lesion type per scan. To this aim, we computed the median, minimum and maximum of the absolute value of the difference between the ground truth percentage of each lesion type obtained from radiologists' annotations and the estimated ones, on the 14 available FAS of the IGR dataset. Third, we evaluated to what extent the segmentation model reproduces the analysis reported by radiologists. To this aim, we first compared the binary decision 'presence or absence of a lesion type' of the network to the radiologist report considered as ground truth. A lesion type was detected by the segmentation model when its estimated volumetry, averaged over both lungs, was above a certain threshold. The difference was then evaluated in terms of detection accuracy and F1 score, for two threshold values, using all scans of the IGR dataset (Supplementary Table 7). Then, we compared disease extent as evaluated by radiologists to the one predicted by the neural network (Supplementary Fig. 3).

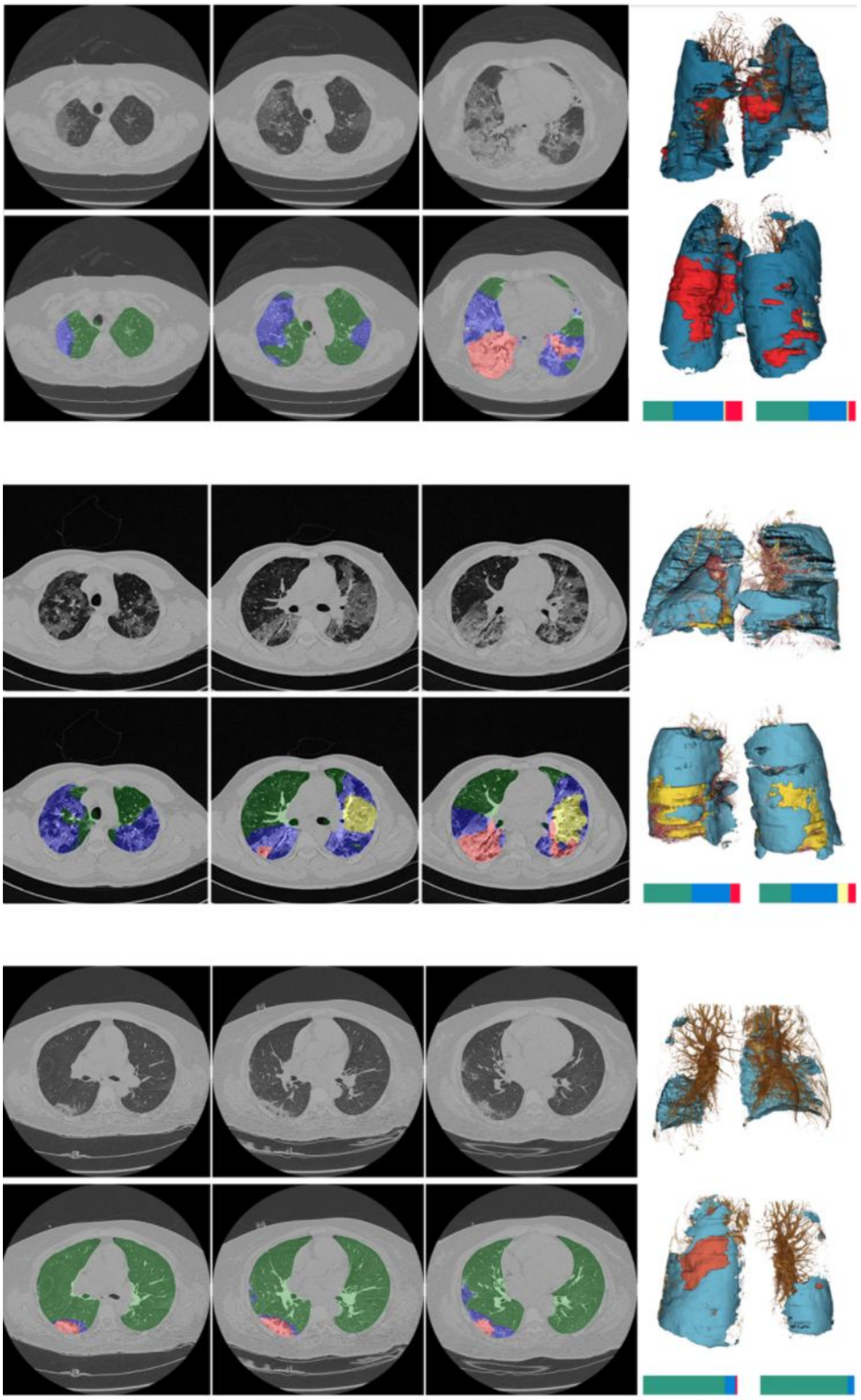

Supplementary Fig. 10: Axial chest CT scans and segmentation results. COVID-19 radiology patterns, as provided by the neural network model for segmentation, for 3 patients with COVID-19. Green/transparent: sane lung; blue: GGO; yellow : crazy paving; red: consolidation. (Top) Patient with diffuse distribution, and multiple large regions of subpleural GGO with consolidation to the right and left lower lobe. Estimated disease extent by AI: 69%/47% (right/left). Radiologist report: critical stage of COVID-19 (stage 5). (Middle) Patient with diffuse distribution and multiple large regions of subpleural GGO with superimposed intralobular and interlobular septal thickening (crazy paving). Estimated disease extent by AI: 51%/68% (right/left). Radiologist report: severe stage of COVID-19 (stage 4). (Bottom) Patient with minimal impairment, and multiple small regions of subpleural GGO with consolidation to the right lower lobe. Estimated disease extent 13%/7% (left/right). Radiologist report: moderate stage of COVID-19 (stage 2).

	GGO	Crazy paving	Consolidation
Accuracy (1% thresh.)	0.7951	0.7684	0.6167
F1 Score (1% thresh.)	0.8848	0.6452	0.7473
Accuracy (2% thresh.)	0.7876	0.7692	0.6667
F1 Score (2% thresh.)	0.8800	0.6182	0.7848

Supplementary Table 7: Detection accuracy and F1 scores of the segmentation model when considering the radiologist report as ground truth. The binary decision used to compute the score is "presence or not of a lesion type". Accuracy and F1 score are averaged over the IGR validation set. We compared, for each patient of the IGR validation set, detection obtained using AI-*segment* to the information provided in the standardized radiologist report. When using the neural network, a lesion type is considered as present when its relative volume with respect to the full volume of both lungs, is above a certain threshold indicated into parenthesis in the 1st column of the table.

**Variable**	**Center**	**Odds ratio (95% lower - 95% upper)**	**P-value**	**P-value Stouffer**
GGO AI	KB	0.61 (0.51,0.73)	3.57e-08	1.37e-08
GGO AI	IGR	0.77 (0.54,1.10)	0.15	
Crazy Paving AI	KB	1.60 (1.29,1.99)	1.74e-05	7.10e-06
Crazy Paving AI	IGR	1.31 (0.92,1.87)	0.13	
Consolidation AI	KB	1.51 (1.27,1.79)	2.85e-06	1.32e-06
Consolidation AI	IGR	1.27 (0.89,1.82)	0.19	
Disease extent AI	KB	2.15 (1.77,2.60)	7.90e-15	1.92e-16
Disease extent AI	IGR	1.90 (1.30,2.79)	9e-4	

Supplementary Table 8: Association between severity and amount of lesions inferred by the segmentation model. For disease extent, we consider the proportion of lung volume. For the other three variables (GGO, consolidation, crazy paving), we normalize them by disease extent so that each variable measures the proportion of the corresponding lesion. Associations with severity are evaluated with logistic regression and reported with 2-sided p-values for each center and p-values were combined with Stouffer's method.

Segmentation results for CT-scans of hospitalized COVID-19 patients

The segmentation model provides automatic quantification of the volume of lesions, expressed as a percentage of the full lung volume (Supplementary Fig. 10). These patterns included the three distinguishable features that appear as disease severity progresses: ground glass opacity (GGO), crazy paving, and finally consolidation. The model was trained on 184 patients from KB hospital (8 fully annotated scans, 176 partially annotated ones) and evaluated on 145 patients from IGR hospital (14 fully annotated scans and 131 partially annotated ones). To evaluate the segmentation network, we first compared its performance to that of radiologists manual annotation. The segmentation network discriminated lung regions from regions outside of the lung with an accuracy of 99.9% when evaluated on the fully annotated scans. Within the lung, the model's ability to discriminate between lesions and healthy areas had F1 values of 0.85 and 0.98 on partially and fully annotated scans. In the fully annotated scans, the predicted volumes of each lesion type had relative errors (median [min-max]) of 3.77% [0.054%-14%] for GGO, 0.96% [0.058%-4.4%] for consolidation, and 5.92% [0.41%-13%] for sane lung (no crazy paving was present in these scans). We next compared the segmentation network to the information contained in the radiology reports. The F1 score measuring the ability of the network to detect the presence of a lesion type per patient, was of 0.88 for GGO, 0.65 for crazy paving, and 0.75 for consolidation (Supplementary Table 7). Correlation between quantification of the proportion of lesions with the network and the radiologist evaluation was of 0.56 (Supplementary Fig. 3). Inspection of visual results were also consistent with radiologist observations (Supplementary Fig. 10 for three representative cases). We lastly evaluated to what extent the segmentation network provided biomarkers of future severity (Supplementary Table 8). We found that severity was significantly associated to GGO extent (OR KB = 0.61 (0.51,0.73), OR IGR = 0.77 (0.54,1.10), $P_{Stouffer}$ = 1.37e-08), crazy paving extent (OR KB = 1.60 (1.29-1.99), OR IGR = 1.31 (0.92,1.87), $P_{Stouffer}$ = 7.10e-06), consolidation extent (OR KB = 1.51 (1.27,1.79), OR IGR = 1.27 (0.89,1.82), $P_{Stouffer}$ = 1.32e-06) as well as total disease extent (OR KB = 2.15 (1.77,2.60), OR IGR = 1.90 (1.30,2.79), $P_{Stouffer}$ = 1.92e-16) (accounting for multiple testing).

Supplementary References

1. Baldeon Calisto, Maria, and Susana K. Lai-Yuen. 2020. “AdaEn-Net: An Ensemble of Adaptive 2D-3D Fully Convolutional Networks for Medical Image Segmentation.” *Neural Networks: The Official Journal of the International Neural Network Society* 126 (June): 76–94.
2. Chen, Sihong, Kai Ma, and Yefeng Zheng. 2019. “Med3D: Transfer Learning for 3D Medical Image Analysis.” *arXiv [cs.CV]*. arXiv. http://arxiv.org/abs/1904.00625.
3. Hara, K., H. Kataoka, and Y. Satoh. 2017. “Learning Spatio-Temporal Features with 3D Residual Networks for Action Recognition.” In *2017 IEEE International Conference on Computer Vision Workshops (ICCVW)*, 3154–60.
4. Ronneberger, Olaf, Philipp Fischer, and Thomas Brox. 2015. “U-Net: Convolutional Networks for Biomedical Image Segmentation.” In *Medical Image Computing and Computer-Assisted Intervention – MICCAI 2015*, 234–41. Springer International Publishing.
5. Yang, Jinzhong, Harini Veeraraghavan, Samuel G. Armato 3rd, Keyvan Farahani, Justin S. Kirby, Jayashree Kalpathy-Kramer, Wouter van Elmpt, et al. 2018. “Autosegmentation for Thoracic Radiation Treatment Planning: A Grand Challenge at AAPM 2017.” *Medical Physics* 45 (10): 4568–81.
