# Supplementary material for: Integrating deep learning CT-scan model, biological and clinical variables to predict severity of COVID-19 patients
Source: Nat Commun. 2021 Jan 27;12:634. doi: 10.1038/s41467-020-20657-4 (PMC7840774; doi:10.1038/s41467-020-20657-4)
Supplement: Supplementary file 3 — Reporting Summary [file 41467_2020_20657_MOESM3_ESM.pdf]

nature research

Corresponding author(s): Michael Blum

Last updated by author(s): Jun 30, 2020

Reporting Summary

Nature Research wishes to improve the reproducibility of the work that we publish. This form provides structure for consistency and transparency in reporting. For further information on Nature Research policies, see our Editorial Policies and the Editorial Policy Checklist.

Statistics

For all statistical analyses, confirm that the following items are present in the figure legend, table legend, main text, or Methods section.

n/a	Confirmed	
☐	☒	The exact sample size (n) for each experimental group/condition, given as a discrete number and unit of measurement
☒	☐	A statement on whether measurements were taken from distinct samples or whether the same sample was measured repeatedly
☐	☒	The statistical test(s) used AND whether they are one- or two-sided<br>*Only common tests should be described solely by name; describe more complex techniques in the Methods section.*
☐	☒	A description of all covariates tested
☐	☒	A description of any assumptions or corrections, such as tests of normality and adjustment for multiple comparisons
☐	☒	A full description of the statistical parameters including central tendency (e.g. means) or other basic estimates (e.g. regression coefficient) AND variation (e.g. standard deviation) or associated estimates of uncertainty (e.g. confidence intervals)
☐	☒	For null hypothesis testing, the test statistic (e.g. F, t, r) with confidence intervals, effect sizes, degrees of freedom and P value noted<br>*Give P values as exact values whenever suitable.*
☒	☐	For Bayesian analysis, information on the choice of priors and Markov chain Monte Carlo settings
☒	☐	For hierarchical and complex designs, identification of the appropriate level for tests and full reporting of outcomes
☐	☒	Estimates of effect sizes (e.g. Cohen's d, Pearson's r), indicating how they were calculated

Our web collection on statistics for biologists contains articles on many of the points above.

Software and code

Policy information about availability of computer code

Data collection

We collected the data by editing spreadsheets manually for clinical and biological data. Variables from the radiologist report were automatically extracted using optical character recognition.

Data analysis

The source code to run all the models of the study (including benchmarks)are open sourced on https://github.com/owkin/scancovia.
Python was used for the analysis along with the following packages:
numpy>=1.18.4, pandas>=0.24.2, SimpleITK==1.2.4, scikit-image>=0.15.0, torch>=1.4.0, torchvision>=0.5.0, nibabel>=2.4.1, tqdm>=4.32.2, scikit-learn>=0.23.1, xgboost>=1.1.1, shap>=0.34.0, tensorflow==1.14, git+https://git@github.com/JoHof/lungmask@master, git+https://git@github.com/lukemelas/EfficientNet-PyTorch@master
Figures were generated using the R package ggplot2 version 3.3.2.

For manuscripts utilizing custom algorithms or software that are central to the research but not yet described in published literature, software must be made available to editors and reviewers. We strongly encourage code deposition in a community repository (e.g. GitHub). See the Nature Research guidelines for submitting code & software for further information.

Data

Policy information about availability of data

All manuscripts must include a data availability statement. This statement should provide the following information, where applicable:

- Accession codes, unique identifiers, or web links for publicly available datasets
- A list of figures that have associated raw data
- A description of any restrictions on data availability

The database of n=1,003 patients from Kremlin-Bicêtre (KB) and Institut Gustave Roussy (IGR) is stored on a server at Institut Gustave Roussy (IGR). The database is available from the first author upon request subject to ethical review. No other database was used.

Field-specific reporting

Please select the one below that is the best fit for your research. If you are not sure, read the appropriate sections before making your selection.

- [x] Life sciences
- [ ] Behavioural & social sciences
- [ ] Ecological, evolutionary & environmental sciences

For a reference copy of the document with all sections, see nature.com/documents/nr-reporting-summary-flat.pdf

Life sciences study design

All studies must disclose on these points even when the disclosure is negative.

Sample size	A database of n=1,003 patients from Kremlin-Bicêtre (KB) and Institut Gustave Roussy (IGR) was collected. This cohort contains all the COVID patients in these institutions from 2020/02/12 to 2020/03/20 for KB and from 2020/03/02 to 2020/04/24 at IGR.
Data exclusions	Two individuals were excluded from the analysis because they explicitely asked to be excluded. Children and pregnant women were excluded from the study.
Replication	Models presented in the study were trained on n=646 patients from KB and evaluated on two independant cohort of n=150 patients from KB and n=135 patients from IGR. Code is available online to reproduce the experiments on new cohorts (https://github.com/owkin/scancovia).
Randomization	Patients in the KB cohort were randomized between a training and a validation set with a stratification on age and outcome.
Blinding	The entire database was anonymized

Reporting for specific materials, systems and methods

We require information from authors about some types of materials, experimental systems and methods used in many studies. Here, indicate whether each material, system or method listed is relevant to your study. If you are not sure if a list item applies to your research, read the appropriate section before selecting a response.

Materials & experimental systems

n/a	Involved in the study
[x]	[ ] Antibodies
[x]	[ ] Eukaryotic cell lines
[x]	[ ] Palaeontology and archaeology
[x]	[ ] Animals and other organisms
[ ]	[x] Human research participants
[x]	[ ] Clinical data
[x]	[ ] Dual use research of concern

Methods

n/a	Involved in the study
[x]	[ ] ChIP-seq
[x]	[ ] Flow cytometry
[x]	[ ] MRI-based neuroimaging

Human research participants

Policy information about studies involving human research participants

Population characteristics	All population characteristics are described in Table 1.
Recruitment	Inclusion criteria were (1) date of admission at hospital (from 2020/02/12 to 2020/03/20 for KB and from 2020/03/02 to 2020/04/24 at IGR) and (2) a positive diagnosis of COVID-19. As IGR is a cancer-research institute, many patients (85%) had cancer compared to KB (7%) or the overall population, making this external cohort more challenging for validation.
Ethics oversight	This study has received approval of ethic committees from the two hospitals (Kremlin Bicêtre Hospital, APHP, Paris and Gustave Roussy Hospital, Villejuif). and authors submitted a declaration to the National Commission of Data Processing and Liberties (N° INDS MR5413020420, CNIL) in order to get registered in the medical studies database and respect the General Regulation on Data Protection (RGPD) requirements.

Note that full information on the approval of the study protocol must also be provided in the manuscript.
