# Supplementary material for: Integrating deep learning CT-scan model, biological and clinical variables to predict severity of COVID-19 patients
Source: Nat Commun. 2021 Jan 27;12:634. doi: 10.1038/s41467-020-20657-4 (PMC7840774; doi:10.1038/s41467-020-20657-4)
Supplement: Supplementary file 4 — Description of Additional Supplementary Files [file 41467_2020_20657_MOESM4_ESM.docx]

**Description of Supplementary Files**

**File Name: Supplementary Data 1**

**Description:** AUC and p-values of the 11 severity scores and of AI-severity, which are used to obtain Figure 2.
